# Laryngeal Mask Ventilation during Neonatal Resuscitation: A Case Series

**DOI:** 10.3390/children9060897

**Published:** 2022-06-16

**Authors:** Lauren White, Katelyn Gerth, Vicki Threadgill, Susan Bedwell, Edgardo G. Szyld, Birju A. Shah

**Affiliations:** 1 Pediatrics, University of Oklahoma (OU) College of Medicine, Oklahoma City, OK 73104, USA; lauren-white@ouhsc.edu (L.W.); katelyn-gerth@ouhsc.edu (K.G.); edgardo-szyld@ouhsc.edu (E.G.S.); 2 Comanche County Memorial Hospital, Lawton, OK 73505, USA; vicki.threadgill@ccmhhealth.com; 3 Fran and Earl Ziegler College of Nursing, OU Health Sciences Center, Oklahoma City, OK 73117, USA; susan.bedwell@ouhealth.com; 4 Oklahoma Children’s Hospital at OU Health, 1200 North Everett Drive, 7th Floor North Pavilion ETNP #7504, Oklahoma City, OK 73104, USA

**Keywords:** laryngeal mask, newborn, resuscitation

## Abstract

Positive pressure ventilation via a facemask is a critical step in neonatal resuscitation but may be a difficult skill for frontline providers or trainees to master. A laryngeal mask is an alternative to endotracheal intubation for some newborns who require an advanced airway. We present the first case series in the United States in which a laryngeal mask was successfully utilized during resuscitation of newborns greater than or equal to 34 weeks’ gestation following an interdisciplinary quality improvement collaborative and focused training program.

## 1. Introduction

Effective delivery of positive-pressure ventilation (PPV), usually initiated via face masks, is a critical step in neonatal resuscitation [1]. However, facemask ventilation is a difficult skill to master, often complicated by leaks around the mask, airway obstructions, and insufficient ventilating pressure [2]. Persistent bradycardia and respiratory depression are indications for ventilatory corrective steps and consideration of alternative airways, such as endotracheal tubes (ETT) or laryngeal masks (LM) [3].

Neonatal endotracheal intubation requires considerable training and experience, which poses a human-factor challenge [4]. An LM is an alternative-airway device that can provide effective PPV, reducing the need for endotracheal intubation while decreasing ventilation time for newborns greater than or equal to 34 weeks’ gestation and 2000 g [5]. However, insufficient experience and training limit LM use and frontline providers report low confidence with LM insertion in real life [6]. Here, we describe our experience following a multistep interdisciplinary effort through a case series of neonates where LM ventilation was successfully utilized.

## 2. Methods

We implemented a three-step LM interdisciplinary collaborative at Oklahoma Children’s Hospital at OU Health (OCH). The goals of this project were to increase availability in LM devices, incorporate LM placement into established neonatal resuscitation training programs, and promote awareness of LM as a safe and effective alternative airway for neonatal resuscitation. The first step in the collaborative effort was to evaluate the pre-existing knowledge and identify barriers to LM use in our center using an anonymous online questionnaire distributed to all Neonatal Resuscitation Program (NRP) trained providers who routinely respond to newborn deliveries. Shah et al. found a low level of confidence in LM placement reported by respondents with the most frequently cited barriers, including limited experience, insufficient training, preference for ETT, and lack of awareness [6].

After analyzing the survey results, an interdisciplinary group was formed to lead the intervention phase. The educational portion of the project focused on teaching when and how to place a LM. Providers were asked to watch a brief skills video provided by the American Academy of Pediatrics [7] and then practice LM insertion during hands-on simulation training with a manikin which took less than two minutes. Front-line newborn resuscitation personnel including residents and registered nurses (RN) were reminded to provide facemask ventilation with corrective steps, as per current NRP guidelines. Then, if the newborn did not positively respond (rise in heart rate after 60 s of initial steps and ventilation), they were instructed to utilize the LM as the alternative interface while simultaneously activating the advanced neonatal resuscitation team. In addition, this group was able to ensure availability of LM in all labor and delivery suites and update hospital LM placement guidelines to include nurses and respiratory therapists.

This is a retrospective chart review of clinical cases in which a LM was utilized as an interface for PPV during neonatal resuscitation performed by OCH providers between 1 September 2020 and 31 December 2021. Eligible patients included infants 34^0/7^ or more weeks’ gestation, both inborn and outborn, who were admitted at OCH, a regional tertiary care center and Level IV Neonatal Intensive Care Unit (NICU), or Comanche County Memorial Hospital (CCMH), a Level II NICU staffed by OCH providers. Cases were reported by verbal or written communication to the study team. The Institutional Review Board (IRB) at the University of Oklahoma Health Sciences Center and CCMH approved the study (IRB #14150) and provided a waiver of consent based on the characteristics of this study. Once eligible cases were identified, the electronic medical record system was reviewed for relevant patient information related to delivery, resuscitation, indication for LM placement, duration of the procedure, respiratory outcomes, clinical disposition, and length of hospital stay.

## 3. Results

We identified ten cases in which a LM was utilized in neonatal resuscitation during the 15-month time period following a quality improvement collaborative. The LM was used as an alternative airway during delivery room resuscitation in four cases at each of our queried units (eight cases total). The demographics and clinical characteristics of these eight patients are presented in Table 1. Of note, a size-1 non-inflatable supraglottic airway, i-gel^®^ (Intersurgical, Wokingham, Berkshire, UK), was the LM device utilized in all but one case (Case 8), in which a size-1 LM with silicone cuff, LMA Unique™ (The Laryngeal Mask Company Limited, Le Rocher, Victoria, Mahe, Seychelles), was used. Additionally, a LM was placed during resuscitation of a newborn requiring transport to OCH from an outside facility and once as part of a code event on a patient with a difficult airway in the NICU. These two cases (Case 9 and Case 10) are described separately below. Placement of the LM provided immediate stabilization in all patients without any noted complications. LM insertion was successful on first attempt in nine cases. Reinsertion was indicated in Case 2 (Table 1) after copious secretions inducing the newborn’s gag reflex led to dislodgement of the initial LM. Three out of the ten patients avoided NICU admission, and all survived to hospital discharge.

### 3.1. Case 9

A 2723 g, 35^6/7^-week gestation male infant was born at a community hospital via vaginal delivery with epidural anesthesia. He was vigorous at birth, and no resuscitation was required. At 5 h of life, the infant had a presumed aspiration event with oxygen desaturation and respiratory distress. The infant was placed on a high-flow nasal cannula with supplemental oxygen at an 8 L per minute flow rate and transfer to a higher level of care was requested. Upon arrival of the neonatal transport team, comprised of two RNs, the infant was noted to have apneic episodes. Intubation was attempted but was unsuccessful. A LM was placed without complication, and the infant was transported, receiving PPV via LM and transport ventilator, to the Level IV NICU at OCH. Upon arrival at the NICU, he was endotracheally intubated and placed on conventional mechanical ventilation. The patient was extubated on day 2 and subsequently transitioned to room air by day 4 of life. The hospital course was complicated by unilateral testicular torsion requiring an orchiectomy. The total hospital stay was 9 days.

### 3.2. Case 10

A 3050 g, 4-month-old, male infant, born at 23^0/7^-weeks gestational age, corrected to 41^3/7^-weeks with a complex medical history, including bronchopulmonary dysplasia, multiple failed tracheal extubation attempts, and glottic granulation tissue removal, suffered an aspiration event on day 129 of life resulting in a neonatal code event. Four attempts at endotracheal intubation (two by a NICU fellow and two by an attending) were unsuccessful, as evidenced by lack of heart rate improvement, presumably due to a visualized laryngospasm preventing ETT insertion and adequate ventilation. Chest compressions and two doses of intravenous epinephrine were administered for a heart rate less than 60 beats per minute, and the emergency airway team was paged to the bedside. A LM was placed by a neonatal nurse practitioner (NNP), resulting in an improvement in heart rate and oxygen saturation. After stabilization, the infant was sedated and successfully intubated by an otolaryngologist. His NICU course was complicated by many comorbidities leading to placement of tracheostomy and gastrostomy tubes and transfer to a children’s rehabilitation center at 203 days of life.

## 4. Discussion

Herein, we document the first ten cases in which a LM was successfully placed by a variety of healthcare providers (physicians, nurses, and trainees) following a quality improvement and educational collaborative at Oklahoma’s regional academic tertiary care center. The authors feel this project was a success based on the presented results as well as verbal feedback from key stakeholders. Minimal effort was required to add LM-specific training to pre-existing newborn resuscitation curricula but dramatically improved awareness of LM as an alternative PPV interface for the frontline practitioner to use when other modalities were not working or available. Safety and effectiveness of LM utilization was demonstrated by improved Apgar scores and immediate clinical outcome or stabilization until endotracheal intubation. While post-resuscitation blood gases are routinely obtained for NICU-admitted patients following advanced resuscitation, these objective markers were not available for all cases in this study.

A systematic review by Cochrane et al. eloquently demonstrates the multitude of difficulties in translating what is known from scientific research to clinical practice. The top barriers to healthcare provider acceptance or adherence to change are grouped into categories, such as cognitive-behavioral, attitudinal, professional, support, and system more so than the lack of convincing evidence [8]. The attitude that an ETT is superior to LM or the professional barrier requiring physician trainees to focus on mastering intubation skills [9,10] would be difficult to overcome. The authors believe we have addressed many of these barriers, as also identified in the survey portion of the project, by improving LM training and awareness, increasing provider confidence in placement, and addressing organizational hurdles related to job description and availability of the device through our collaborative effort. These ten cases represent a small but significant change in practice at our hospitals, as our goal was not to replace facemask ventilation or endotracheal intubation but to increase awareness of LM as a rescue PPV interface. Universal adherence to choosing LM as the first alternative airway when initial ventilation has failed was not expected owing to our well-developed protocol to access the advanced neonatal resuscitation team and the availability of in-house providers experienced in endotracheal intubation.

LM use for newborn resuscitation has been a topic of interest in recent neonatal resuscitation literature. Singular published case reports document successful LM insertion and ventilation after the failure of tracheal intubation and facemask PPV in infants with micrognathia and other congenital airway anomalies, such as tracheal stenosis and those found in Cornelia de Lange syndrome, Smith-Lemli-Opitz syndrome, and Pierre-Robin sequence [11,12,13,14,15]. A large randomized controlled non-inferiority trial in Uganda conducted on the safety of LM insertion in the hands of providers with limited experience and concluded that LM was comparable to facemask ventilation for asphyxiated newborns [16]. However, the generalizability of these findings toward the industrialized world may be limited, mainly because the study was performed among unsupervised midwifes using the LM as primary resuscitative device. In addition, the characteristics of the patients presented in the referred study include a higher proportion of neonates with meconium-stained or foul-smelling amniotic fluid and very early neonatal death which may not represent the typical population in high-resource settings. Ease of insertion, efficacy as a rescue ventilation device, and safety when compared to endotracheal intubation in specific populations of newborns are less in question, but a paucity of data to support the adoption of LM as the preferred device during neonatal resuscitation still exists in developed countries, leading to difficulties in implementation [5,17,18,19].

A new systematic review with treatment recommendations from the International Liaison Committee on Resuscitation (ILCOR) Neonatal Life Support Task Force suggests that a LM can be used in place of facemask for PPV in newborns greater than or equal to 34 weeks’ gestation when resources and training permit. Several knowledge gaps have been identified, including the training required for successful insertion as well as the effectiveness and safety in certain populations [20]. Our case series demonstrates the effectiveness and safety of LM for PPV in infants at least 34 weeks’ gestation in the desperate situation of “cannot ventilate, cannot intubate” as well as the limited training required to achieve successful placement in real-life scenarios. The use of LM for PPV among newborn infants less than 34 weeks’ gestation or as the primary ventilation device in newborn resuscitation remains to be studied in larger trials.

We recognize that our study is an observational retrospective chart review limited to frontline providers from a single academic institution practicing at two hospitals. However, to our knowledge, this is the first clinical case series in which LM was utilized to provide advanced neonatal resuscitation in the United States. We contend that our results will encourage continued use and empower newborn resuscitation providers at our center and others with the evidence that LM is a safe and effective tool.

## 5. Conclusions

LM can be used as a safe and effective airway interface for resuscitation and/or stabilization of late preterm and term neonates in the delivery room, in the NICU, or during transport. We demonstrate that frontline providers successfully utilized LM following an interdisciplinary quality improvement collaborative and focused training program.

## Figures and Tables

**Table 1:** Characteristics of Infants Receiving Laryngeal Mask Intervention in the Delivery Room.

	Case 1	Case 2	Case 3	Case 4	Case 5	Case 6	Case 7	Case 8
Location	OCH	OCH	OCH	OCH	CCMH	CCMH	CCMH	CCMH
Perinatal history; Mode of delivery	Scant PNC; SVD	Repeat C/S	Category 2 FHT, Meconium; C/S for FTD	Twin gestation; scheduled C/S	Category 3 FHT; unscheduled C/S	Category 3 FHT; unscheduled C/S	Fetal distress	Late PNC, Substance use
Gestational age (weeks)	36^1/7^	38^1/7^	37^3/7^	38^0/7^	39^1/7^	37^2/7^	36^6/7^	39^0/7^
Birthweight (grams)	3455	3140	3210	3070	4510	2790	3560	2570
Apgar Score (1, 5, 10, 15 min)	1, 9	7, 8	1, 4, 6, 8	1, 6	2, 2, 3	0, 5, 6	7, 6, 7	3, 4, 7, 8
Providers present	RN	RN/Resident	Resident/ NNP	Resident	RN/NNP	RN/NNP	RN/NNP	RN/NNP/ Anesthesiologist
Indication for LM	Apnea, Failure of facemask PPV	Persistent grunting, retractions with facemask CPAP	Non-vigorous, poor response to facemask PPV	Failure of facemask PPV	No response to facemask PPV	Respiratory failure	Poor respiratory effort	Respiratory failure
LM device used	i-gel^®^	i-gel^®^	i-gel^®^	i-gel^®^	i-gel^®^	i-gel^®^	i-gel^®^	LMA^®^
Placement time after birth	2 min 59 s	18 min 9 s, 19 min 40 s	4 min 40 s	3 min	2 min	n/a	12 min	4 min
Number of attempts	1	2	1	1	1	1	1	1
LM duration	n/a	58 s	4 min	2–3 min	4 min	4 min	3 min	3 min
Immediate outcome	HR > 100/min, improved perfusion, spontaneous breathing	Crying, coughing, normalized respiratory effort	Improved oxygen saturations	HR, saturations, and color improved	HR and saturations improved	n/a	HR and saturations improved	HR and saturations improved
Max FiO2	n/a	0.6	0.8	n/a	1	n/a	1	1
Respiratory outcome/support	Spontaneous breathing	Spontaneous breathing	Spontaneous breathing/ CPAP	Stable on room air by 15 min	Intubated	Intubated	Intubated	Weaned to NIV
Disposition	MBU	MBU	NICU	MBU	TOC to Level 4 NICU	TOC to Level 4 NICU	TOC to Level 4 NICU	NICU
Length of hospital stay (days)	2	2	4	3	13	10	10	6

CCMH = Comanche County Memorial Hospital (Level II NICU); CPAP = Continuous Positive Airway Pressure; C/S = Cesarean Section; FHT = Fetal Heart Tracing; FiO2 = Fraction of Inspired Oxygen; FTD = Failure to Descend; HR = Heart Rate; LM = Laryngeal Mask; MBU = Mother-Baby Unit; n/a = not available or reported; NICU = Neonatal Intensive Care Unit; NIV = Non-invasive Ventilation; NNP= Neonatal Nurse Practitioner; OCH = Oklahoma Children’s Hospital (Level IV NICU); PNC = Prenatal Care; PPV = Positive Pressure Ventilation; RN = registered nurse; SVD = Spontaneous Vaginal Delivery; TOC = Transfer of Care.

## Data Availability

The de-identified data presented in this study are available on request from the corresponding author. The data are not publicly available due to compliance with HIPAA.
